# Spatiotemporal Analysis of Developing Brain Networks

**DOI:** 10.3389/fninf.2018.00048

**Published:** 2018-07-31

**Authors:** Ping He, Xiaohua Xu, Han Zhang, Gang Li, Jingxin Nie, Pew-Thian Yap, Dinggang Shen

**Affiliations:** ^1^Department of Computer Science, Yangzhou University, Yangzhou, China; ^2^Department of Radiology and BRIC, University of North Carolina, Chapel Hill, NC, United States; ^3^School of Psychology, South China Normal University, Guangzhou, China; ^4^Department of Brain and Cognitive Engineering, Korea University, Seoul, South Korea

**Keywords:** structural correlation networks, developmental networks, cortical thickness, developmental meta-network decomposition, non-negative matrix factorization

## Abstract

Recent advances in MRI have made it easier to collect data for studying human structural and functional connectivity networks. Computational methods can reveal complex spatiotemporal dynamics of the human developing brain. In this paper, we propose a Developmental Meta-network Decomposition (DMD) method to decompose a series of developmental networks into a set of Developmental Meta-networks (DMs), which reveal the underlying changes in connectivity over development. DMD circumvents the limitations of traditional static network decomposition methods by providing a novel exploratory approach to capture the spatiotemporal dynamics of developmental networks. We apply this method to structural correlation networks of cortical thickness across subjects at 3–20 years of age, and identify four DMs that smoothly evolve over three stages, i.e., 3–6, 7–12, and 13–20 years of age. We analyze and highlight the characteristic connections of each DM in relation to brain development.

## 1. Introduction

Understanding normal brain development is critical for basic developmental neuroscience. Recent advances in MRI have made it easier to collect data for studying human structural and functional connectivity networks. For example, the multi-site NIH MRI Study of Normal Brain Development has collected developmental structural MRI of over a thousand of children, ranging from infancy to young adulthood. The rich spatial and temporal information afforded in such datasets calls for sophisticated computational methods to capture the dynamic changes in brain connectivity during the course of development.

To date, most developmental studies are mainly focused on charting network characteristics, such as small-world properties, global efficiency, local efficiency, and etc. (Menon, 2013; Olde Dubbelink et al., 2013; Wu et al., 2013; Hadley et al., 2016). They treat the developmental networks as a group of static networks and compute their network characteristics independently. Alternatively, community detection algorithms are used to investigate the temporal evolution of modular organization (Mucha et al., 2009; Betzel and Bassett, 2017). The detected modules are recognized as sets of brain regions that perform specific cognitive functions.

To study temporal changes in brain connectivity, various matrix decomposition methods, especially principal component analysis (PCA), independent component analysis (ICA), and non-negative matrix factorization (NMF) have been applied to developmental structural and functional networks to identify the intrinsic components (Ghanbari et al., 2014; Sotiras et al., 2017) or connectivity states (Leonardi et al., 2013; Calhoun et al., 2014; Miller et al., 2016). Compared with other matrix decomposition methods, NMF is featured with its non-negativity constraint imposed on the elements of the decomposed factor matrices. This constraint leads to a parts-based representation (Lee and Seung, 1999), thus making the decomposition results more interpretable. During brain development, brain connectivity may evolve through different stages. Accordingly, the intrinsic connectivity components, which we refer to as meta-networks, may also develop across different stages. In this paper, we call such evolving intrinsic component as *developmental meta-networks* (DMs) and propose an approach for Developmental Meta-network Decomposition (DMD). DMD decomposes the developmental networks into a set of temporally smooth DMs that capture the underlying connectivity patterns across developmental stages to adapt to the cognitive development. DMD not only automatically identifies the developmental stages and the number of DMs, but also uncovers the evolution of DMs. In this study, we apply DMD to *developmental networks* constructed based on inter-regional cortical thickness correlation across subjects for age spanning 3–20 years.

## 2. Materials and methods

### 2.1. Datasets and construction of blue developmental networks

This study uses the Pediatric MRI Data Repository^1^ released by the NIH MRI Study of Normal Brain Development (Evans, 2006), which is a multi-site developmental study with the objective to collect developmental data for investigating brain maturation in connection with behavioural and cognitive development in normal populations (Evans, 2006; Waber et al., 2007). We adopt 951 scans from 445 subjects of 3 to 20 years of age (Figure S1). The age in years are obtained by subtracting the date of birth from the date of visit. Each subject is scanned in two or more MRI sessions over a five-to-six-year period.

For each participant, a three-dimensional sagittal T1-weighted image was acquired using a 1.5 T scanner with 1 mm isotropic resolution. Each image was skull stripped to remove non-cerebral tissues (Smith, 2002), corrected for intensity inhomogeneity, and segmented into gray matter (GM), white matter (WM), and cerebrospinal fluid (CSF) (Zhang et al., 2001). FSL 4.3 were used to perform brain extraction (BET) and tissue segmentation (FAST). To ensure the image processing quality, we visually checked the brain extraction and segmentation results for each subjects less than 5 years old. Different parameters were tried until the segmentation result were acceptable. Inner and outer cortical surfaces were reconstructed by a deformable surface method (Li et al., 2012). With the automated anatomical labelling (AAL) template (Tzourio-Mazoyer et al., 2002), each cortical surface was parcellated into 78 regions of interest (ROIs) (see Table S1) based on a non-linear hybrid volumetric/surface registration method (Liu et al., 2004).

Cortical thickness was measured between the reconstructed inner and outer cortical surfaces using the shortest distance in the native space at each vertex (Liu et al., 2008). Regional cortical thickness was defined as the average thickness of all vertices within an ROI. We removed the effects of multiple confounding variables, including gender and overall mean cortical thickness, via linear regression analysis for each cortical region at each age (He et al., 2007). The regression residuals were taken as the corrected cortical thickness values.

The details of networks construction based on cortical thickness is described in Nie et al. (2013). An inter-regional cortical thickness correlation network was built for each time point by computing the pairwise Pearson's correlation coefficient of cortical properties across subjects. Take age 3 for example, there were 45 unique subjects, then each ROI was represented by a cortical thickness vector of length 45. By computing the Pearson's correlation coefficient between each pair of ROI vectors, we built a 78 × 78 inter-regional correlation network for age 3. We used the absolute values of the correlation matrices as in other studies (Khundrakpam et al., 2013). In the network matrix, a zero entry represents absence of connection, whereas a positive entry indicates the strength of pairwise inter-regional correlation. In this way, we obtained the developmental networks composed of eighteen 78 × 78 symmetric non-negative networks of 3–20 years of age (Figure S2).

### 2.2. Developmental meta-network decomposition (DMD)

**Figure 1:**
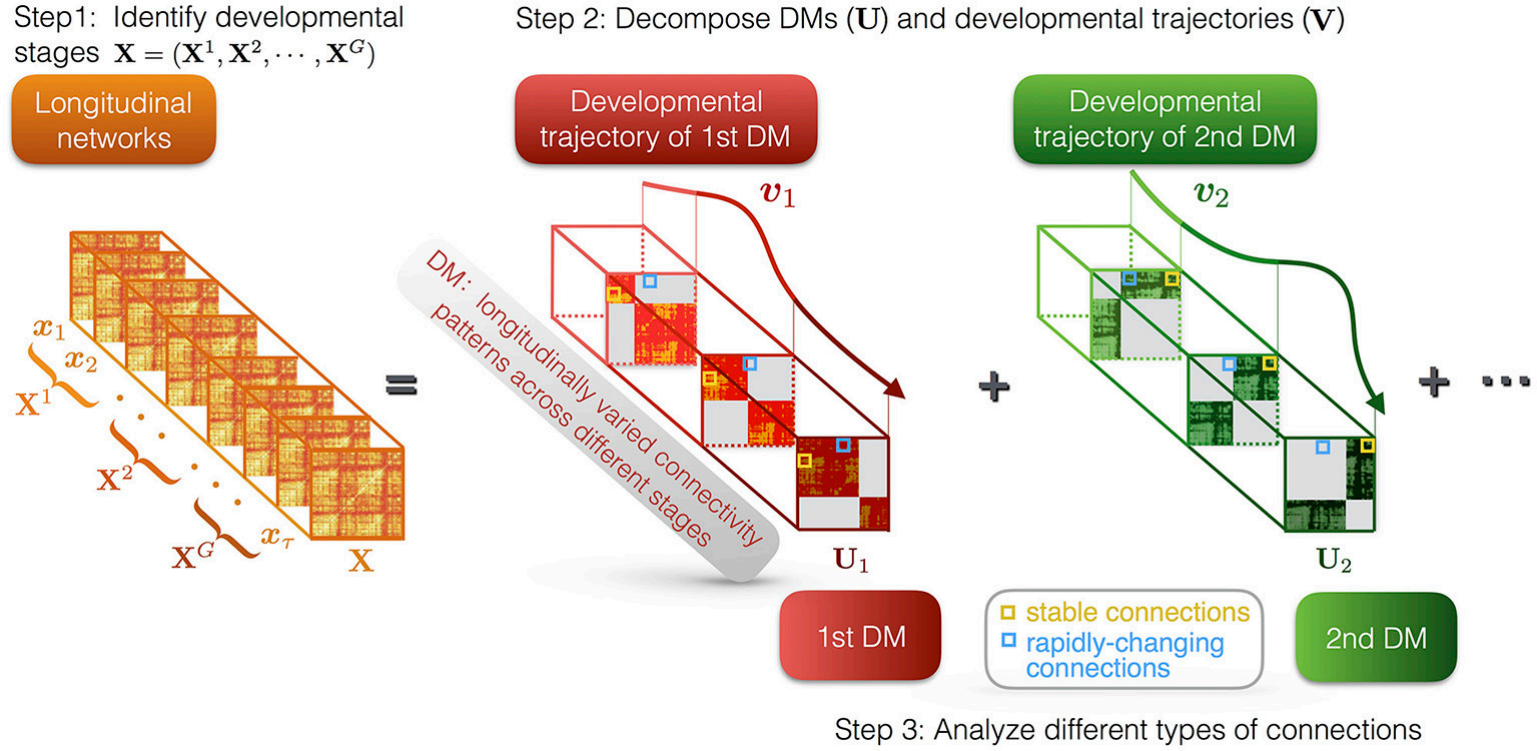
Illustration of DMD method. First, we identify *G* developmental stages of the longitudinal networks *X*, which are denoted as **X**^1^, **X**^2^, ⋯ , *X*^*G*^. Second, DMD decomposes the longitudinal networks into a set of DMs (*U*) and their developmental trajectories (*V*). For instance, *U*_1_ and *U*_2_ respectively represent the first and the second DM, while ***v***_1_ and ***v***_2_ represent their developmental trajectories. The DMs reveal the developmental connectivity patterns, while the developmental trajectories indicate the longitudinal contributions of the DMs. Each DM has different states at different stages. Third, we characterize different types of connections in DMs, i.e., stable and rapidly-changing connections.

DMD method is composed of three parts (see Figure 1): (i) identifying the developmental stages of the developmental networks; (ii) decomposing the developmental networks into a set of DMs and their trajectories; (iii) characterization of the different types of connections in DMs, i.e., stable and rapidly-changing connections.

#### 2.2.1. Identifying developmental stages

The evolution of DMs is essentially caused by the change of developmental networks across different developmental stages. An ideal partition of developmental stages satifies that the networks in the same stage are close to each other, while the networks in different stages are significantly different from each other.

To achieve this aim, we use the method of consensus clustering (Ozdemir et al., 2015; Wu et al., 2015), which yields a stable and robust partition.

We run the sensitive k-means clustering algorithm on the vectorized developmental brain networks with different numbers of clusters. For each number of clusters, we record the clustering results for 100 runs with randomized seeds, and use the criterion of *dispersion coefficient* to evaluate the consistency of the clustering results (Brunet et al., 2004). The larger the dispersion coefficient, the more consistent the clustering results. The optimal partition of developmental stages is the one that leads to the largest dispersion coefficient in the finest granularity. In another word, if there are multiple partitions leading to the same highest dispersion coefficient, we prefer the one that reveals the most detailed developmental stages of the age-related networks.

#### 2.2.2. Developmental meta-network decomposition

Let **X** = [***x***_1_ ***x***_2_ ⋯ ***x***_τ_] be the developmental non-negative networks, where $x_{i}\in {\mathbb{R}}_{+}^{M}$ represents the vectorized network at the *i*^*th*^ time point, *M* is the number of connections in each network (i.e., 78 × 77/2 in this study due to the symmetry of network), τ is the number of time points. Assume that the τ developmental networks consist of *G* developmental stages, let ${\text{X}}^{t}=\left[x_{i_{1}}\;x_{i_{2}}\;\cdots \;x_{i_{n_{t}}}\right]\in {\mathbb{R}}^{M\times n_{t}}$ (∀*t* ∈ [1, *G*]) denote the *t*^*th*^ developmental stage, then **X** can be reorganized as **X** = [**X**^1^ **X**^2^ ⋯ **X**^*G*^]. Our aim is to decompose a set of DMs (denoted as **U**) and their developmental trajectories (denoted as **V**) from **X**. Note that we allow each DM to evolve across different stages, instead of remaining unchanged through development.

We start from considering the decomposition of a single developmental stage **X**^*t*^. It is an observation of the *t*^*th*^ developmental stage that consists of two parts. One is the true developmental networks of the *t*^*th*^ stage (${\hat{\text{X}}}^{t}$), the other is the noise introduced during image acquisition, preprocessing and etc. To reduce the impact of noise, we perform decomposition on ${\hat{\text{X}}}^{t}$ instead of observed **X**^*t*^. We assume that ${\hat{\text{X}}}^{t}$ can be represented as a linear combination of *p* DMs at the *t*^*th*^ stage (**U**^*t*^), whose contributions are indicated by their trajectories (**V**^*t*^), i.e., ${\hat{\text{X}}}^{t}={\text{U}}^{t}{{\text{V}}^{t}}^{T}$. The combined objective function of true data recovery and DM decomposition is given in Equation (1).

(1) $$\begin{array}{l} \underset{{\hat{X}}^{t}}{\min}\;\frac{1}{2}||X^{t}-{\hat{X}}^{t}|{|}_{F}^{2}+\lambda ||{\hat{X}}^{t}|{|}_{*}\\ \text{s}.\text{t}.\;{\hat{X}}^{t}=U^{t}V^{t}{\;}^{T},U^{t}{\;}^{T}U^{t}=I,U^{t}\geq 0,V^{t}\geq 0 \end{array}$$

The first term in Equation (1) minimizes the difference between ${\hat{\text{X}}}^{t}$ and **X**^*t*^, the second term penalizes the nuclear norm of ${\hat{\text{X}}}^{t}$, which implies the number of DMs. The orthogonal constraint **U**^*t*^*T*^^**U**^*t*^ = **I** avoids finding overlapped connectivity patterns. Besides, the non-negativity constraints on **U**^*t*^ and **V**^*t*^ facilitate their neurobiological interpretation as DMs and developmental trajectories. To simplify the formulation, we replace ${\hat{\text{X}}}^{t}$ with the product of **U**^*t*^ and **V**^*t*^*T*^^ in Equation (1) and have the following objective function.

(2) $$\begin{array}{l} \underset{\begin{array}{c} U^{t},V^{t}\geq 0\\ U^{t}{\;}^{T}U^{t}=I \end{array}}{\min}\;\frac{1}{2}||X^{t}-U^{t}V^{t}{\;}^{T}|{|}_{F}^{2}+\lambda ||U^{t}V^{t}{\;}^{T}|{|}_{*} \end{array}$$

Next, we take all the developmental stages into consideration. According to existing neural network models, nervous systems can change smoothly by slowly changing connectivity patterns and strength (Enquist and Ghirlanda, 2005). Inspired by that, we define the overall objective function as the sum of all the single-stage objective function plus a cross-stage smoothness regularization term R(U,V).

(3) $$\begin{array}{l} \underset{\begin{array}{c} U^{t},V^{t}\geq 0\\ U^{t}{\;}^{T}U^{t}=I \end{array}}{\min}\;\sum_{t\;=\;1}^{G}\left(\frac{1}{2}||X^{t}-U^{t}V^{t}{\;}^{T}|{|}_{F}^{2}+\lambda ||U^{t}V^{t}{\;}^{T}|{|}_{*}\right)+R(U,V) \end{array}$$

where

(4) $$\begin{array}{l} R(U,V)\;\underline{\underline{\text{def}}}\;\frac{\alpha }{4}\sum_{k,l\;=\;1}^{G}W_{U}(k,l)||U^{k}-U^{l}|{|}_{F}^{2}+\frac{\beta }{4}\sum_{i,j\;=\;1}^{\tau }W_{V}(i,j)v_{i}-v_{j}{\;}^{2}) \end{array}$$

In Equation (4), the first term of R(U,V) measures the temporal smoothness of *U* across different states, where **W**_**U**_(*k, l*) indicates the adjacency between the *k*^*th*^ and *l*^*th*^ developmental stages. The second term of R(U,V) measures the temporal smoothness of *V* across different time points, where **W**_**V**_(*i, j*) measures the adjacency between the *i*^*th*^ and *j*^*th*^ time points.

Since it has been proved that minimizing the nuclear norm of the product of two non-negative matrices is equivalent to minimizing half of their squared Frobenius norm (Srebro and Shraibman, 2005), we can replace the nuclear norm $||{\text{U}}^{t}{{\text{V}}^{t}}^{T}|{|}_{*}$ in Equation (3) with $\frac{1}{2}\left(||{\text{U}}^{t}|{|}_{F}^{2}+||{\text{V}}^{t}|{|}_{F}^{2}\right)$. Besides, notice that $||{\text{U}}^{t}|{|}_{F}^{2}=\text{tr}\left({{\text{U}}^{t}}^{T}{\text{U}}^{t}\right)=p$ and $\overset{G}{\underset{t=1}{\sum }}||{\text{V}}^{t}|{|}_{F}^{2}=||\text{V}|{|}_{F}^{2}$, we can rewrite the objective function in the following form.

(5) $$\begin{array}{l} J=\underset{\begin{array}{c} U^{t},V^{t}\geq 0\\ U^{t}{\;}^{T}U^{t}=I \end{array}}{\min}\;\sum_{t=1}^{G}\frac{1}{2}||X^{t}-U^{t}V^{t}{\;}^{T}|{|}_{F}^{2}+\frac{1}{2}\lambda ||V|{|}_{F}^{2}+R(U,V) \end{array}$$

Therefore, we obtain the multiplicative updating rules for **U**^*t*^ and **V** as follows.

(6) $$\begin{array}{l} U^{t}\leftarrow U^{t}⊙\frac{\left[X^{t}V^{t}+\alpha \sum_{k=1}^{G}W_{U}(t,k)U^{k}\right]}{\left[U^{t}V^{t}{\;}^{T}X^{t}V^{t}+\alpha \sum_{k=1}^{G}W_{U}(t,k)U^{t}U^{t}{\;}^{T}U^{k}\right]} \end{array}$$

(7) $$\begin{array}{l} V\leftarrow V⊙\frac{\left[\sum_{t=1}^{G}H^{t}X^{t}{\;}^{T}U^{t}+\beta W_{V}V\right]}{\left[\sum_{t=1}^{G}H^{t}H^{t}{\;}^{T}VU^{t}{\;}^{T}U^{t}+\lambda V+\beta D_{V}V\right]} \end{array}$$

where ${\text{H}}^{t}\overset{\text{def}}{=}\left(e_{i_{1}},e_{i_{2}},\cdots \;,e_{i_{n_{t}}}\right)$ is the mask matrix of **V**^*t*^ satisfying **V**^*t*^ =**H**^*t*^*T*^^**V**, ***e***_*i*_*j*__ is a 0/1 vector with the only positive element at the *i*_*j*_-th index, **D**_**V**_ is the degree matrix of **W**_**V**_, ⊙ and the bar respectively denote element-wise product and division. The convergence proof of the multiplicative updating rules can be referred to that of non-negative matrix decomposition (Lee and Seung, 1999). To avoid local minimum, we adopt an initialization strategy on **U** and **V** similar to that of *k*-means algorithm, i.e., repeating multiple (100) times with random initializations and choosing the one with the least cost. The empirical convergence of DMD method is shown on Figure S3. The method is robust to a wide range of the regularization parameters (Figure S4).

The number of DMs is determined by maximizing Minimal Trajectory Distance (MTD), which computes the minimal distance between pairwise developmental trajectories. The larger MTD, The more separable the developmental trajectories, the more distinct and biologically significant the corresponding DMs.

#### 2.2.3. Characterizing different types of connections in DMs

On the basis of developmental meta-network decomposition, we characterize two types of connections in DMs, i.e., *stable connections* and *rapidly-changing connections*, for analysis.

The *stable connections* constitute the “bones” of DMs that keep unchanged through development, while their contributions in the developmental networks are indicated by the developmental trajectories. We select the stable connections with the least normalized divergence (e.g., top 2%) for each DM, and exclude those insignificant connections with average strength ($\bar{{\text{U}}_{r}}$) less than a threshold (δ_*r*_). Take the *r*^*th*^ DM (denoted as **U**_*r*_) for example,

(8) $$\begin{array}{l} \Omega (U_{r})=\frac{\text{div}(U_{r})}{\bar{U_{r}}}=\frac{\left[\sum_{k,l=1}^{G}\;u_{r}^{k}-u_{r}^{l}\right]}{\left[\frac{1}{G}\sum_{t=1}^{G}u_{r}^{t}\right]} \end{array}$$

Ω(**U**_*r*_) in Equation (8) computes the normalized divergence of the connections in the the *r*^*th*^ DM, where $u_{r}^{t}$ represents the *t*^*th*^ state of **U**_*r*_. The threshold δ_*r*_ is adaptively determined by the quadratic mean (or root mean square value) of **U**_*r*_, where *GM* is the number of connections in **U**_*r*_.

(9) $$\begin{array}{l} \delta_{r}=\sqrt{\frac{{\left\|U\right\|}_{rF}^{2}}{GM}} \end{array}$$

The *rapidly-changing connections* highlight the most important changes in the inter-regional coordination during brain development. We characterize this type of connections by taking into account of both DMs and their developmental trajectories. For each DM, we select the most significantly increased and decreased connections across adjacent stages by computing $\Delta u_{r}^{t}=\left(u_{r}^{t+1}-u_{r}^{t}\right)$. Meanwhile, we calculate the stage contributions of each DM by averaging its within-stage trajectory, i.e., ${\overset{-}{v}}_{r}^{t}=\text{mean}\left(v_{r}^{t}\right)$. The rapidly-changing connections are the ones with the same direction of change (↑ or ↓) in both DMs ($\Delta u_{r}^{t}$) and their stage contributions across adjacent stages ($\Delta {\overset{-}{v}}_{r}^{t}={\overset{-}{v}}_{r}^{t+1}-{\overset{-}{v}}_{r}^{t}$), thus indicating the changes in the developmental networks.

#### 2.2.4. Reproducibility

To evaluate the reproducibility of DMD method, we use a split-half strategy, i.e., dividing the developmental networks into two halves with odd-numbered ages (3, 5, ⋯ , 19) and even-numbered ages (4, 6, ⋯ , 20). The reproducibility is quantified by computing similarity between the corresponding DMs (or developmental trajectories), which are independently decomposed from the two splits and matched using the Hungarian algorithm (Kuhn, 2005). We use the cosine similarity to measure the similarity between the matched DMs (or trajectories), as did in Lange et al. (2004). Since the two splits of developmental networks have a temporal gap of one year, cosine similarity is especially advantageous in the evaluation of trajectory reproducibility, because it is a judgment of orientation instead of magnitude.

## 3. Results

We apply DMD method to the developmental structural correlation networks and obtain four DMs, which evolve across three identified developmental stages, including ages 3–6, 7–12, and 13–20, respectively (Figure S5). For the identification of developmental stages and the DM number, please refer to the subsection *Parameter Influence*.

### 3.1. Stable and rapidly-changing connections of DMs

**Figure 2:**
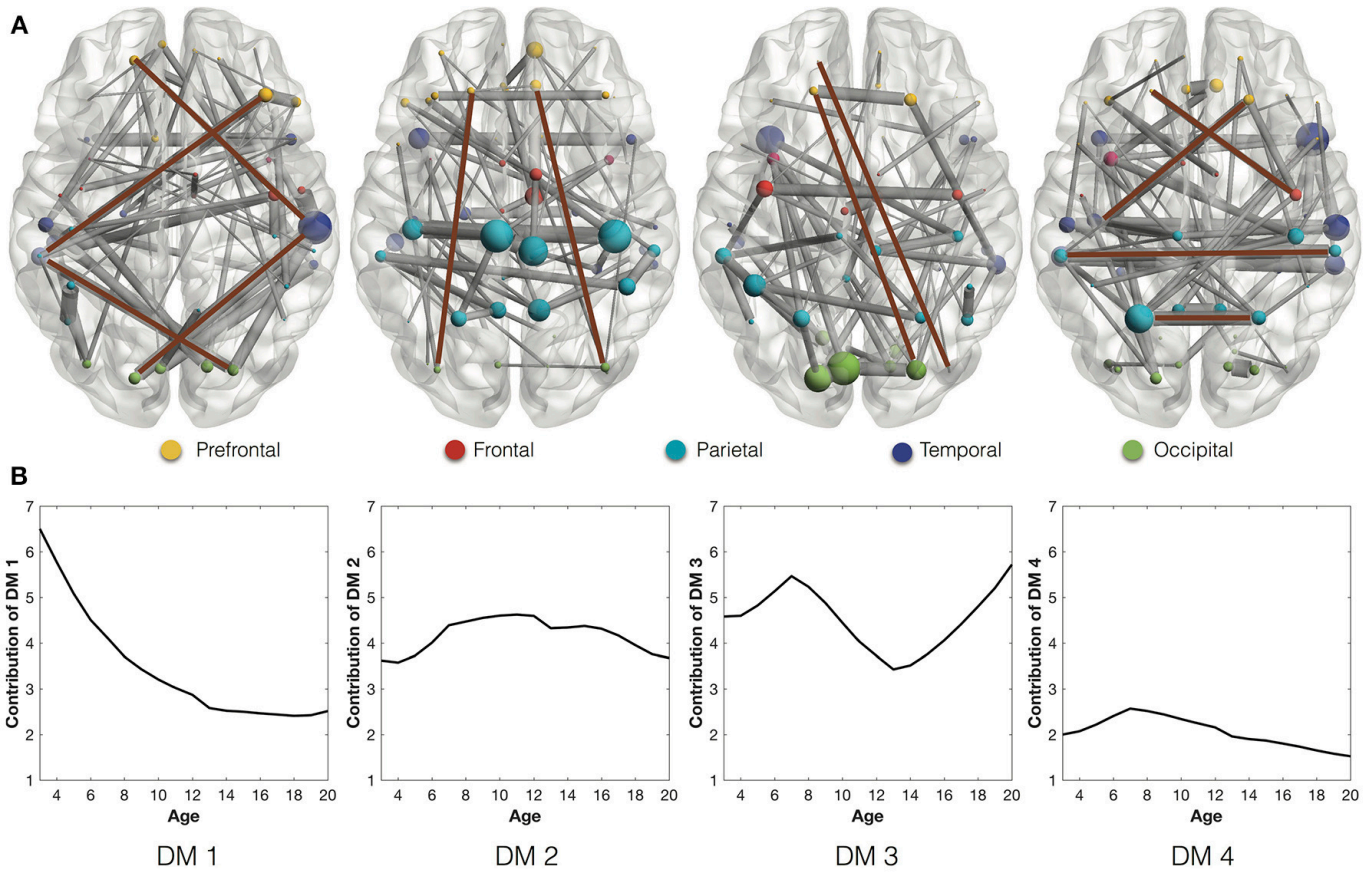
**(A)** Stable connections of the four DMs through development. The brown lines highlight the representative connections. Different ROIs are rendered with different colors according to their anatomical locations (Wang et al., 2007): prefrontal, frontal, parietal, temporal, and occipital. **(B)** The developmental trajectories of the four DMs. They reflect the contributions of the stable connections in the longitudinal networks.

We extract the stable connections of the four DMs (Figure 2A), whose contributions in the development are indicated by the developmental trajectories (Figure 2B). In the first DM, the stable connections exhibit an *indirect connection pattern*, which looks like “<>,” between the prefrontal and occipital areas through interlaying temporal regions. The trajectory of the first DM shows that the *indirect connection pattern* decreases quickly at first but then slows down after the age of 13. In comparison, the stable connections of the second and third DMs are characterized with the *long direct connections* between the prefrontal and occipital areas. The trajectories of these *direct connections*, after going through ups and downs, reach higher values at the age of 20 than at the age of 3 years. Combined together, they indicate the gradual replacement of the *indirect connection pattern* by the *direct connection pattern* from age 3 to 20 years. This is consistent with the development of functional network architecture, where regional interactions change from being predominantly anatomically local in children to interactions spanning longer cortical distances in young adults (Vogel et al., 2010). Besides, the stable connections of the fourth DM are composed of short-range connections and homologous connections between homotopic regions. Similar connections were also found as the most common and robust connections among different individuals (Hermundstad et al., 2013).

**Figure 3:**
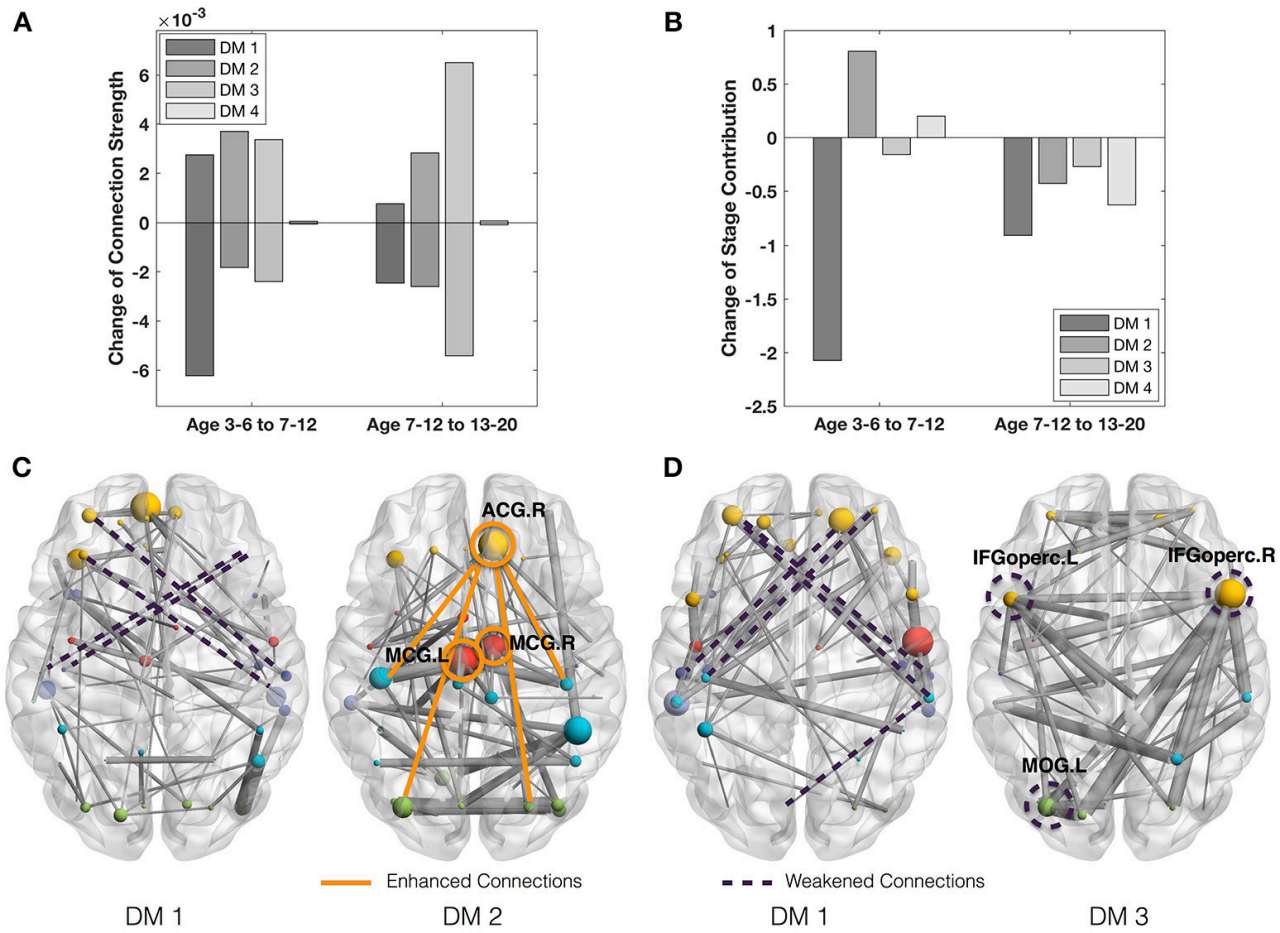
**(A)** Mean strength of the increase and decrease of the connection strength in the four DMs across adjacent states. **(B)** Change of the stage contributions by the four DMs across adjacent states. **(C)** Rapidly-changing connections from ages 3–6 to 7–12. **(D)** Rapidly-changing connections of DMs from ages 7–12 to 13–20.

On the other side, we study the rapidly-changing connections of DMs. Since the fourth DM is the most stable one with almost negligible change of connection strength (Figure 3A), we will focus on the rapidly-changing connections of the other three DMs. During the transition from the first developmental stage (ages 3–6) to the second (ages 7–12), the first DM shows both significantly decreased connection strength and stage contribution (Figures 3A,B). Its rapidly-weakened connections indicate the decrease of the frontal part of the *indirect connection pattern* (<>) between the prefrontal and temporal regions (Figure 3C). Meanwhile, the second DM shows both significantly increased connection strength and stage contribution. Its rapidly-enhanced connections highlight three hubs, ACG.R (right anterior cingulate gyrus) and bilateral MCG (middle cingulate gyrus). Since those regions are involved in emotion formation and processing (Bush et al., 2000; Hadland et al., 2003), their enhanced connections may indicate the *emotional development* after 7 years old. In fact, 7 years of age is indeed the critical time point when impulses of primitive emotion are subjugated to reason and internalized social control (Cole et al., 1994; Pierre Philippot, 2013).

In the transition from the second developmental stage (ages 7–12) to the third (ages 13–20), the first DM is still featured with decreased connection strength and decreased stage contribution (Figure 3D). Its rapidly-weakened connections demonstrate the continued decrease of the frontal part of the *indirect connection pattern* between the prefrontal and temporal regions. Meanwhile, the third DM shows the most significantly decreased connection strength and moderately decreased stage contribution. Its rapidly-weakened connections highlight three inter-connected hubs, including bilateral IFGoperc (opercular inferior frontal gyri) and MOG.L (left middle occipital gyrus). Since IFGoperc.L play a role in processing word phonology (Shaywitz et al., 1995) and IFGoperc.R is involved in visual and auditory spelling tasks (Booth et al., 2003), their weakened connections may indicate the *decline of language development* since 13 years old. This finding is consistent with Lenneberg's classic hypothesis about the age limitation (12–13 years) in the first language acquisition (Lenneberg, 1967) as well as Collier's studies that the first language acquisition is largely completed by the age of 12 (Collier, 1987, 1989).

To summarize, we have three major findings from the characteristic connections of DMs, which are validated in Figure S6.

1. The *indirect connections* between the prefrontal and occipital regions gradually, to some extent, get replaced by the *direct connections* from 3 to 20 years of age.
2. The connections with the *emotion*-related regions (ACG.R and bilateral MCG) are significantly *increased* from 6 to 12 years of age.
3. The connections with the *language*-related regions (IFGoperc.L and IFGoperc.R) are significantly *decreased* from 13 to 20 years of age.

### 3.2. Parameter influence

**Figure 4:**
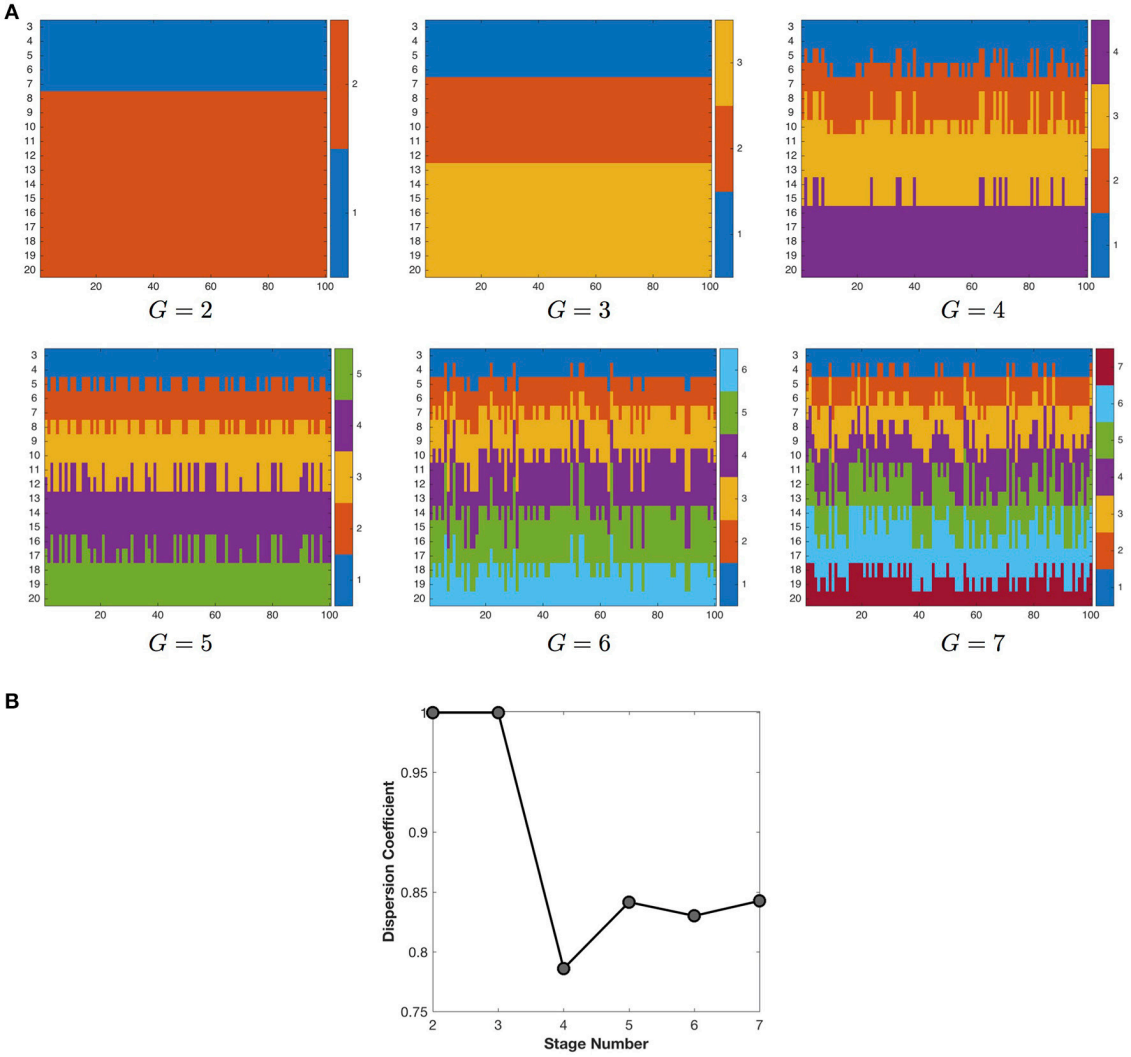
**(A)** The partition of developmental stages under different settings of stage number. Due to the space limit, we only show the partition results for 2–7 developmental stages. The X axis represents the 100 runs of k-means clustering algorithm, the Y axis represents the longitudinal networks at 3–20 years of age. The different colors indicate different stage assignment. **(B)** The influence of stage number on the quality (dispersion coefficient) of the developmental stage partition. The maximal dispersion coefficient 1 indicates the most robust partition of developmental stages.

We first study the influence of stage number on the quality of the developmental stage partition (Figure 4). With the increase of stage number (*G*), the developmental stage partition at first keeps very robust in consensus clustering, but then exhibits growing instability when *G* becomes larger than 3. Therefore, in this study we choose the most robust developmental stage partition in the finest granularity, i.e., three stages including 3–6, 7–12, and 13–20 years of age. Note that although we do not utilize any temporal information, the developmental stage partitions still group the networks at adjacent time points together, indicating the smooth change of the developmental networks.

**Figure 5:**
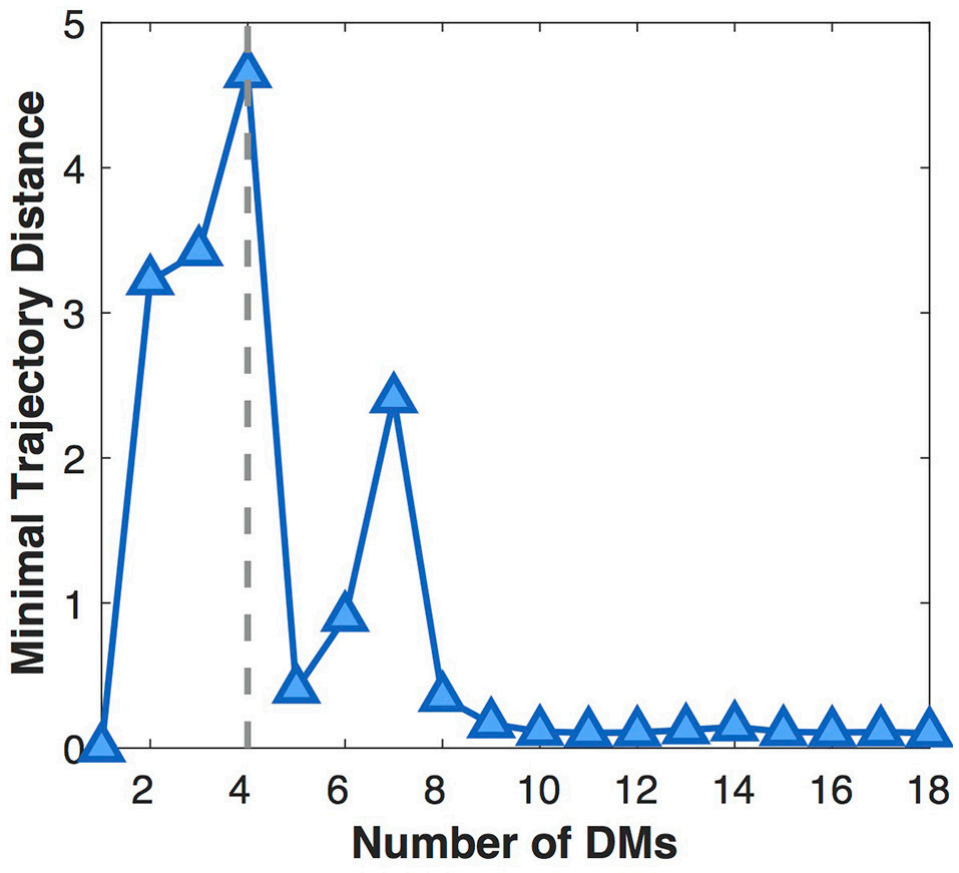
Influence of the DM number on the separability of developmental trajectories (MTD). The larger the MTD value, the more separable the developmental trajectories.

Next we show the influence of the DM number on the separability of developmental trajectories (Figure 5). With the increase of the DM number, MTD at first gradually increases because more and more distinct DMs are separated from the average pattern (underfitting). When the DM number grows larger than four, MTD sharply drops off, then rises to a local maximum at seven DMs, which might indicate the existence of DM sub-patterns. After that, MTD continues to decline and finally reaches a very low value, because more and more insignificant DMs are separated from the dominant ones (overfitting). Therefore, in this study we choose four DMs that leads to the most distinct DMs with the most separable developmental trajectories.

### 3.3. DMD reproducibility

**Figure 6:**
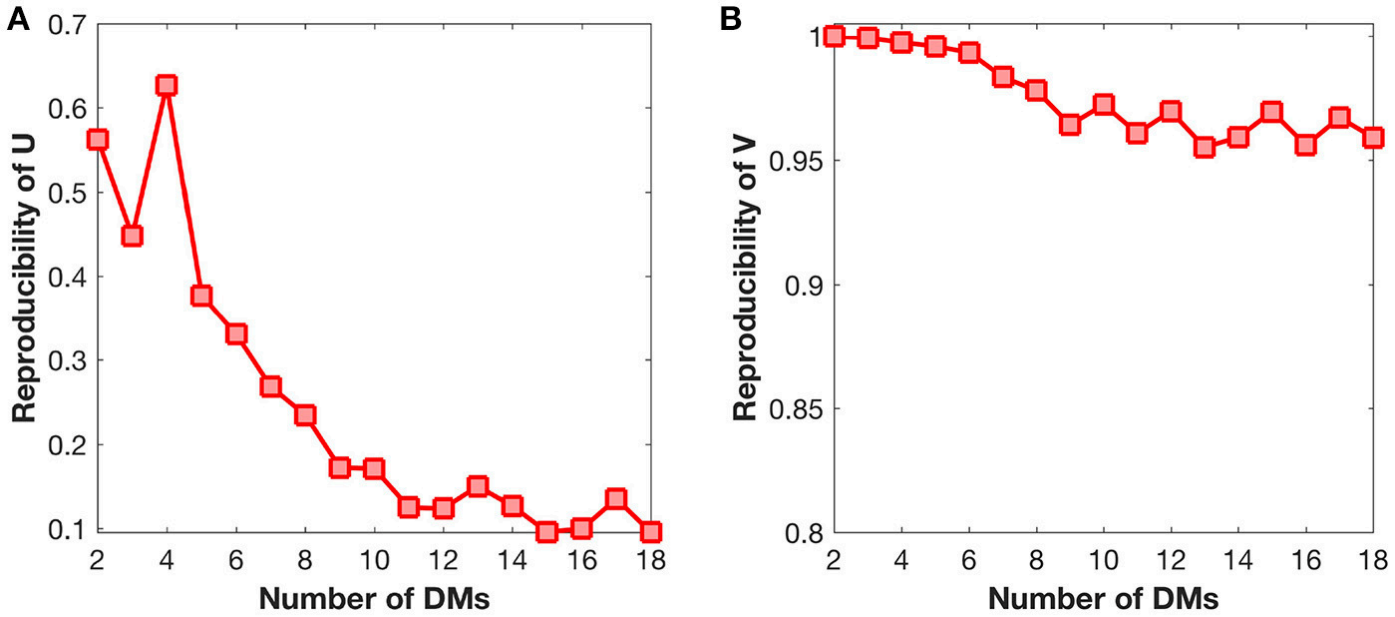
Reproducibility of DMs (*U*) and developmental trajectories (*V*) under different settings of DM number. **(A)** Reproducibility of DMs with the growth of DM number. **(B)** Reproducibility of developmental trajectories with the growth of DM number.

We also examine the reproducibility of DMs and their developmental trajectories under different settings of DM number (Figure 6). The reproducibility of DMs reaches maximum at the DM number of four (Figure 6A), while the reproducibility of developmental trajectories get slightly decreased with the increase of the DM number (Figure 6B). Therefore, in this study it is a reasonable choice to set the DM number as four.

## 4. Discussion

We propose the DMD method to decompose developmental networks into a set of DMs and their developmental trajectories. One important advantage of our approach is that it goes beyond current static network decomposition strategies to capture dynamic temporal patterns of developmental networks. Moreover, it automatically identifies the partition of developmental stages as well as the number of DMs. Our analysis of normal developmental brain networks shows that the decomposed DMs contain not only stable connections but also rapidly-changing connections. Capturing variability in the connectivity patterns via DMs moves us closer to the clinic of the future.

DMD helps to find significant underlying changes in connectivity over development. For instance, the rapidly-changing connections of DMs reveal that (1) the connections with the emotion-related regions (ACG.R and bilateral MCG) are significantly increased after 7 years of age, and (2) the connections with the language-related regions (bilateral IFGoperc) are significantly decreased after 13 years of age. These findings are consistent with the existing behavioral studies that emotion control gets enhanced at the age of 7 years (Cole et al., 1994; Pierre Philippot, 2013) and the first language acquisition is largely completed by the age of 12 years (Lenneberg, 1967; Collier, 1987, 1989). Compared with previous studies, DMD provides more connectome details for the development neuroscience community.

DMD has many other applications. In the clinical domain, DMs allow in-depth comparison between normal and abnormal developmental networks, which are usually related to a certain type of disease. We can apply the normal DMs, which are decomposed from the normal developmental networks, to the abnormal developmental networks and obtain its abnormal developmental trajectories. By comparing the *normal* and *abnormal* developmental trajectories under the same DMs, we are able to identify when and how the abnormal trajectories deviate from the normal ones, thus providing insights into the underlying mechanism of the disease. For example, in this study the first three DMs show that the *indirect connections* between the prefrontal and occipital areas gradually get replaced by the *direct connections* with the growth of age. This indicates the increase of functional efficiency in the normal brain development (Achard and Bullmore, 2007; Vogel et al., 2010; Bullmore and Sporns, 2012), because direct neural connections are generally believed to use less time for signal transmission than the polysynaptic connections (Grossenbacher, 2001). Hence, if the abnormal trajectories of the first three DMs exhibit different trends from the normal ones, it implies the correlation between the disease and the functional efficiency development.

## 5. Limitations and future work

The current DMD method can only be applied to cross-sessional developmental networks, hence fail to fully utilize the longitudinal information of subjects. In our future work, we plan to extend the DMD method for early diagnosis of developmental disease of single subjects. This requires the application of DMD to within-subject, instead of across-subject, longitudinal networks. Although the data are progressively easier to collect based on Diffusion Tensor Imaging (DTI) or Functional MRI (fMRI), the main challenge lies in that different sets of DMs will be derived from different within-subject longitudinal networks. Therefore, it is necessary to extend DMD to decompose a representative set of DMs from multiple within-subject longitudinal networks. Then the representative set of DMs can be applied to single subjects for early disease diagnosis. We believe that DMD method will ultimately pave the way to much more refined representation and understanding of brain network development, diseases, and other longitudinal biological phenomena.

## Ethics statement

This study was carried out in accordance with the recommendations of Name of Guidelines, Name of Committee with written informed consent from all subjects. All subjects gave written informed consent in accordance with the Declaration of Helsinki. The protocol was approved by the Name of Committee.

## Author contributions

PH contributed to designing and analyzing the experiments, summarizing and visualizing the results, and writing the manuscript. XX contributed to developing and implementing the method, visualizing and analyzing the results. HZ, GL, and P-TY contributed to the critical revision of the manuscript and the discussion of the work. JN contributed to preparing the data and revising the manuscript. DS contributed to directing the whole project.

### Conflict of interest statement

The authors declare that the research was conducted in the absence of any commercial or financial relationships that could be construed as a potential conflict of interest.

## Footnotes

^1^https://pediatricmri.nih.gov/nihpd/info/index.html
