# Supplementary material for: Spatiotemporal Analysis of Developing Brain Networks
Source: Front Neuroinform. 2018 Jul 31;12:48. doi: 10.3389/fninf.2018.00048 (PMC6080575; doi:10.3389/fninf.2018.00048)
Supplement: Supplementary file 1 [file Data_Sheet_1.pdf]

Supplementary Material: Spatiotemporal Analysis of Developing Brain Networks

1 SUPPLEMENTARY TABLES AND FIGURES

1.1 Tables

1.2 Figures

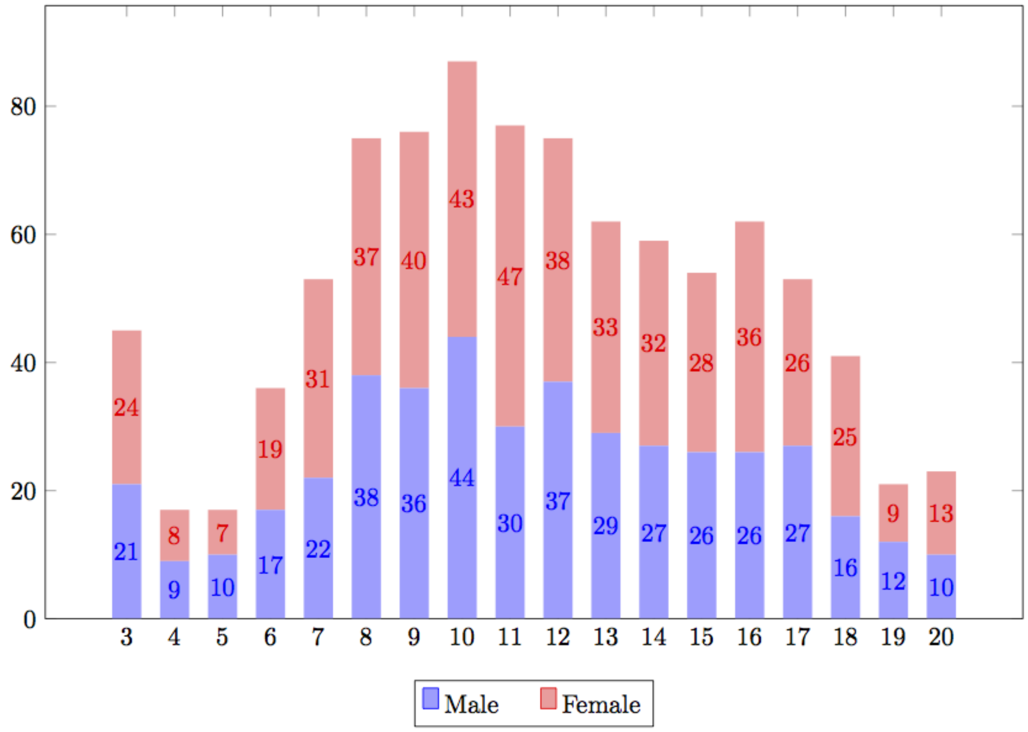

Figure S1. Session distribution of males and females at each ege

Table S1. Summary of 78 Cortical Regions

Region	Abbrev	Region	Abbrev
Precental gyrus	PreCG.L	Precental gyrus right	PreCG.R
Superior frontal gyrus left, dorsolateral	SFGdor.L	Superior frontal gyrus right, dorsolateral	SFGdor.R
Superior frontal gyrus left, orbital part	ORBsup.L	Superior frontal gyrus right, orbital part	ORBsup.R
Middle frontal gyrus left	MFG.L	Middle frontal gyrus right	MFG.R
Middle frontal gyrus left, orbital part	ORBmid.L	Middle frontal gyrus right, orbital part	ORBmid.R
Inferior frontal gyrus left, opercular part	IFGoperc.L	Inferior frontal gyrus right, opercular part	IFGoperc.R
Inferior frontal gyrus left, triangular part	IFGtriang.L	Inferior frontal gyrus right, triangular part	IFGtriang.R
Inferior frontal gyrus left, orbital part	ORBinf.L	Inferior frontal gyrus right, orbital part	ORBinf.R
Rolandic operculum left	ROL.L	Rolandic operculum right	ROL.R
Supplementary motor area left	SMA.L	Supplementary motor area right	SMA.R
Olfactory cortex left	OLF.L	Olfactory cortex right	OLF.R
Superior frontal gyrus left, medial	SFGmed.L	Superior frontal gyrus right, medial	SFGmed.R
Superior frontal gyrus left, medial orbital	ORBsupmed.L	Superior frontal gyrus right, medial orbital	ORBsupmed.R
Gyrus rectus left	REC.L	Gyrus rectus right	REC.R
Insula left	INS.L	Insula right	INS.R
Anterior cingulate and paracingulate gyri left	ACG.L	Anterior cingulate and paracingulate gyri right	ACG.R
Median cingulate and paracingulate gyri left	MCG.L	Median cingulate and paracingulate gyri right	MCG.R
Posterior cingulate gyrus left	PCG.L	Posterior cingulate gyrus right	PCG.R
Hippocampus left	HIP.L	Hippocampus right	HIP.R
Parahippocampal gyrus left	PHG.L	Parahippocampal gyrus right	PHG.R
Amygdala left	AMYG.L	Amygdala right	AMYG.R
Calcarine fissure and surrounding cortex left	CAL.L	Calcarine fissure and surrounding cortex right	CAL.R
Cuneus left	CUN.L	Cuneus right	CUN.R
Lingual gyrus left	LING.L	Lingual gyrus right	LING.R
Superior occipital gyrus left	SOG.L	Superior occipital gyrus right	SOG.R
Middle occipital gyrus left	MOG.L	Middle occipital gyrus right	MOG.R
Inferior occipital gyrus left	IOG.L	Inferior occipital gyrus right	IOG.R
Fusiform gyrus left	FFG.L	Fusiform gyrus right	FFG.R
Postcentral gyrus left	PoCG.L	Postcentral gyrus right	PoCG.R
Superior parietal gyrus left	SPG.L	Superior parietal gyrus right	SPG.R
Inferior parietal left	IPL.L	Inferior parietal right	IPL.R
Supramarginal gyrus left	SMG.L	Supramarginal gyrus right	SMG.R
Angular gyrus left	ANG.L	Angular gyrus right	ANG.R
Precuneus left	PCUN.L	Precuneus right	PCUN.R
Paracentral lobule left	PCL.L	Paracentral lobule right	PCL.R
Caudate nucleus left	CAU.L	Caudate nucleus right	CAU.R
Lenticular nucleus left, putamen	PUT.L	Lenticular nucleus right, putamen	PUT.R
Lenticular nucleus left, pallidum	PAL.L	Lenticular nucleus right, pallidum	PAL.R
Thalamus left	THA.L	Thalamus right	THA.R
Heschl gyrus left	HES.L	Heschl gyrus right	HES.R
Superior temporal gyrus left	STG.L	Superior temporal gyrus right	STG.R
Temporal pole left: superior temporal gyrus	TPOsup.L	Temporal pole right: superior temporal gyrus	TPOsup.R
Middle temporal gyrus left	MTG.L	Middle temporal gyrus right	MTG.R
Temporal pole left: middle temporal gyrus	TPOmid.L	Temporal pole right: middle temporal gyrus	TPOmid.R
Inferior temporal gyrus left	ITG.L	Inferior temporal gyrus right	ITG.R

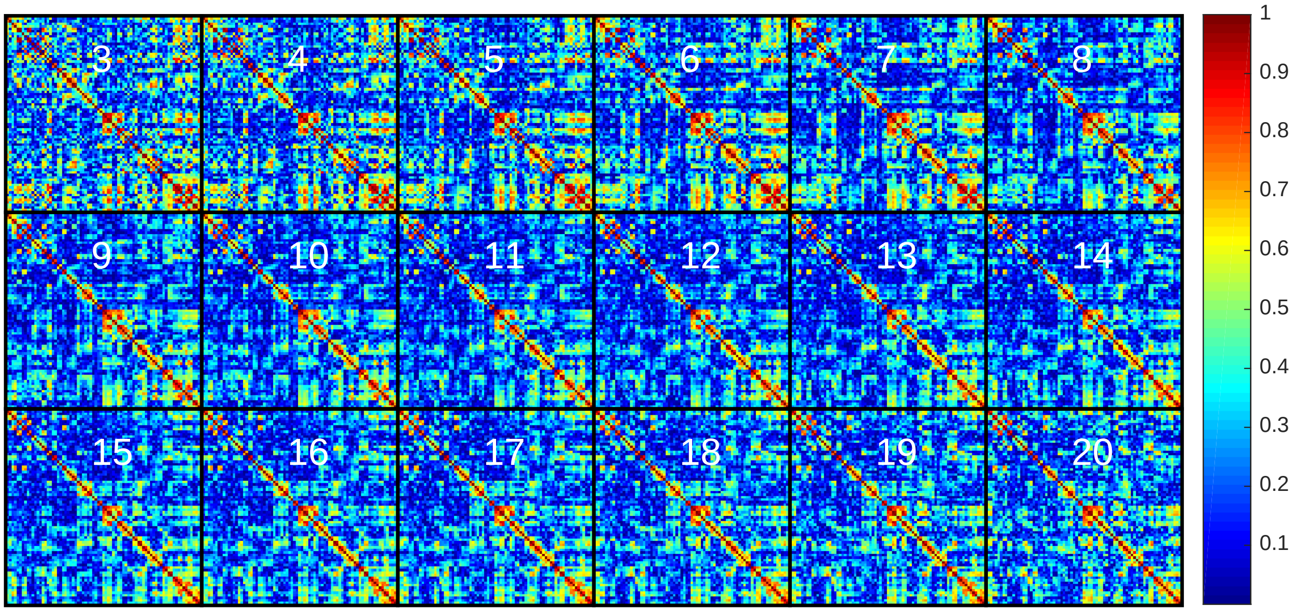

Figure S2. Longitudinal correlation networks based on cortical thickness From Age 3 to 20

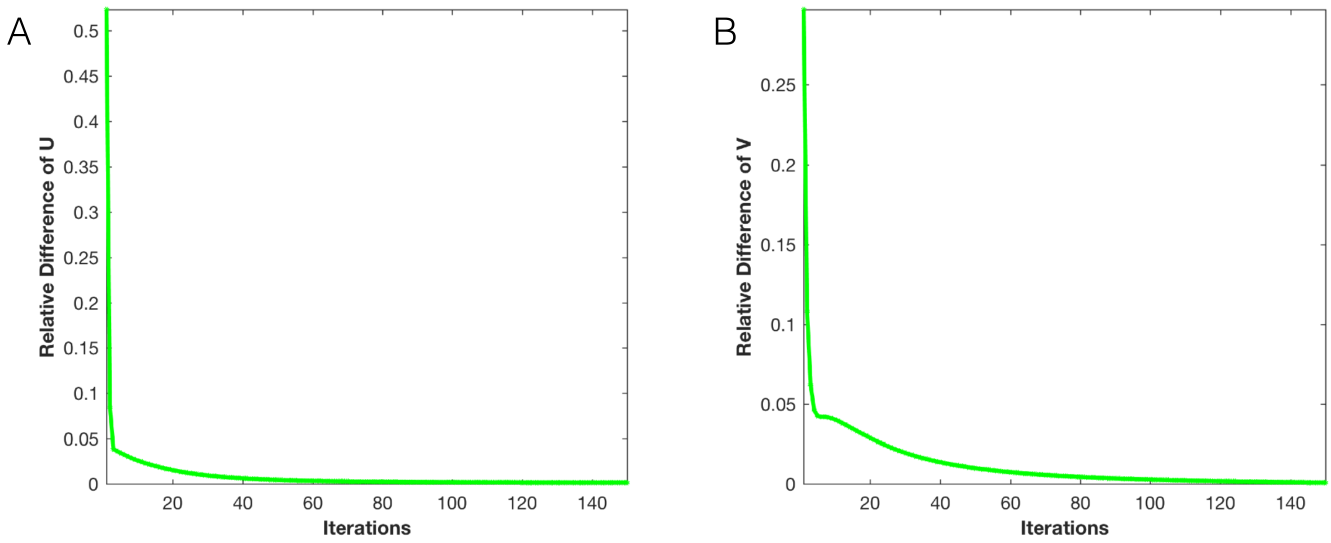

Figure S3. Convergence performance of DMD method. (A) The relative difference of $\mathbf{U}$ during consecutive iterations ($\frac{\|\mathbf{U}(i+1)-\mathbf{U}(i)\|}{\|\mathbf{U}(i)\|}$), where $\mathbf{U}(i)$ represents the DMs ($\mathbf{U}$) obtained in the ith iteration. (B) The relative difference of $\mathbf{V}$ during consecutive iterations ($\frac{\|\mathbf{V}(i+1)-\mathbf{V}(i)\|}{\|\mathbf{V}(i)\|}$), where $\mathbf{V}(i)$ represents the developmental trajectories ($\mathbf{V}$) obtained in the ith iteration. DMD converges after a few iterations.

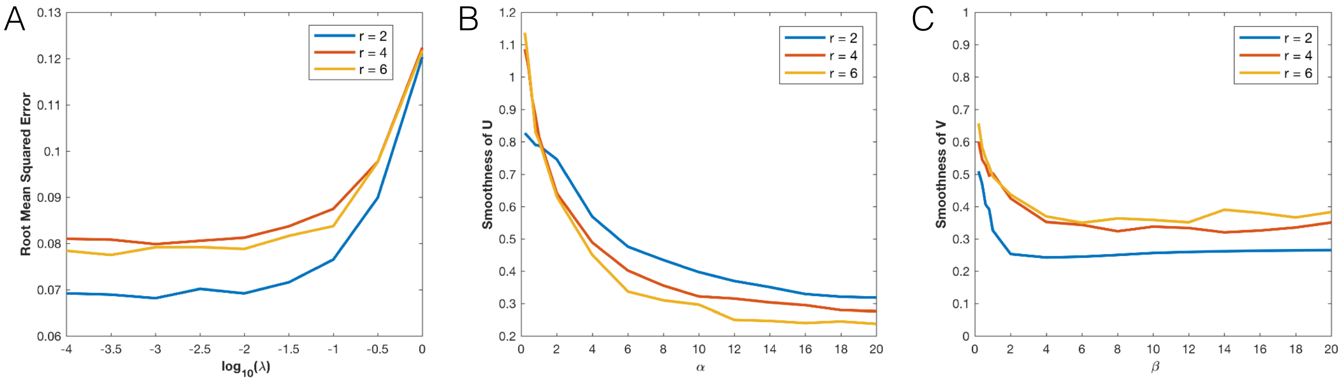

Figure S4. Sensitivity test of the regularization parameters. (A) Influence of the parameter λ on the reconstruction error (root mean squared error) with fixed $\alpha = 12$, $\beta = 2$ and various DM numbers (r). (B) Influence of the parameter α on the smoothness of DM ($\frac{1}{G-1}\sum_t \frac{\|\mathbf{U}^{t+1}-\mathbf{U}^t\|}{\|\mathbf{U}^t\|}$) with fixed $\beta = 2$, $\lambda = 0.01$ and various DM numbers. (C) Influence of the parameter β on the smoothness of developmental trajectories ($\frac{1}{G-1}\sum_t \frac{\|\mathbf{V}^{t+1}-\mathbf{V}^t\|}{\|\mathbf{V}^t\|}$) with fixed $\alpha = 12$, $\lambda = 0.01$ and various DM numbers. DMD is robust in a wide range of parameter settings ($\lambda \leq 0.01$, $\alpha \geq 12$ and $\beta \geq 2$).

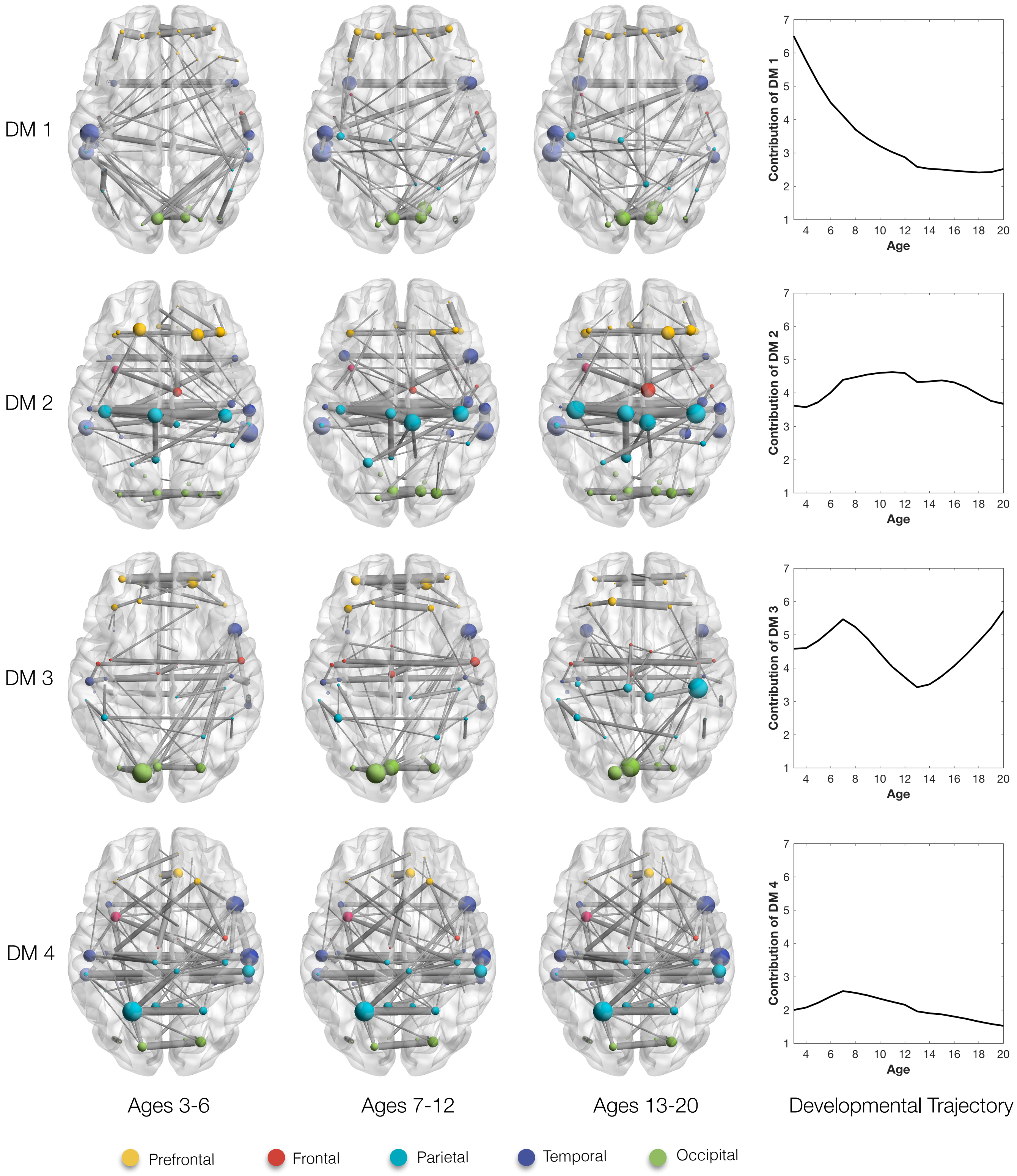

Figure S5. Overview of four DMs. The top 2% connections are illustrated for visualization.

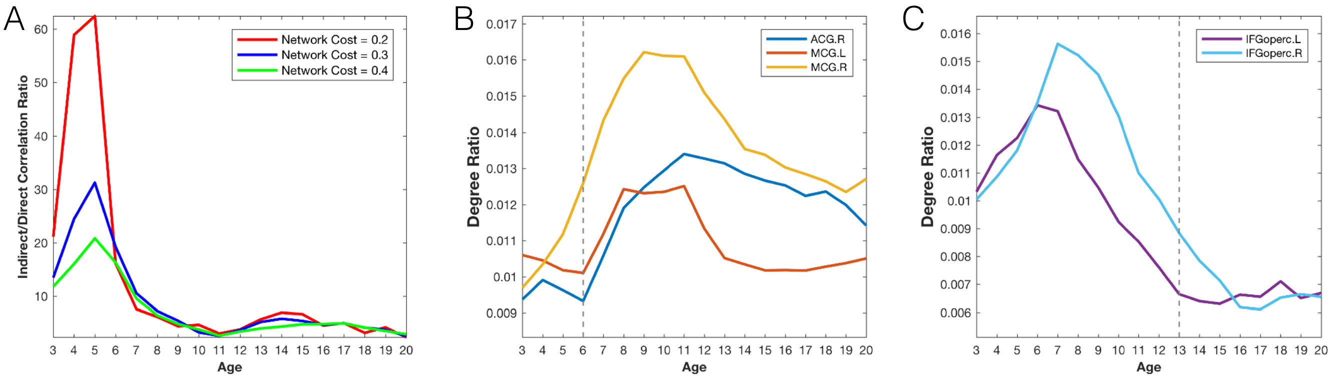

Figure S6. Validation of the major findings in the longitudinal networks. (A) The indirect/direct connection ratio between the prefrontal and occipital regions generally declines with the growth of age. The network cost refers to the ratio of the number of reserved edges in a network to the maximum possible number of pair-wise connections (78×78). The lower network cost leads to the stronger reserved correlations. (B) The degree ratios of the emotion-related regions (ACG.R and bilateral MCG) significantly increase during the age span of 7-12 years. The significance of one-tail t-test for these three regions, compared with the previous age span of 3-6 years, is $p < 0.001$. (C) The degree ratios of the language-related regions (IFGoperc.L and IFGoperc.R) significantly decrease during the age span of 13-20 years. The significance of one-tail t-test for these two regions, compared with the previous age span of 7-12 years, is $p < 0.001$.
